# Physiological Action of Crystallized Aconitine

**Published:** 1872-08-01

**Authors:** 


### PHYSIOLOGICAL ACTION OF CRYSTALLIZED ACONITINE.

Duquesnel, the distinguished French Pharmacist, has recently published, in the *Journal de Pharmacie et de Chimie*, an article on the active principle of aconite.

The "aconitine" obtained by chemists hitherto, with one exception, has been, according to Duquesnel, an amorphous article of uncertain strength and composition. The true, active principle of aconite, is crystallized aconitine, as obtained by him.—[See Duquesnel's process in the *Chicago Pharmacist* for July.]

The following article of MM. Grahant and Duquesnel, which we translate from the *Journal de Pharmacie et de Chimie*, contains a highly interesting demonstration of the physiological effects of true aconitine.

For the purpose of studying the physiological action of crystallized aconitine, we prepared a solution containing 1 milligramme to the cubic centimetre of water, and made the following series of experiments on the frog:

*First experiment:* We injected under the skin of the back of a frog by means of a Pravaz syringe, $\frac{1}{2}$ of a milligramme (8-10,000 of a grain) of aconite. At first the animal was agitated, the head drooped forward on the thorax; thirty minutes after the injection of this feeble dose of poison, the exposed sciatic nerve had completely lost its motor excitability; whilst the muscles of the thigh acted promptly under induced currents. Upon laying open the thorax, the heart was observed to beat regularly.

*Second experiment:* The Gastrocnemius muscle of the frog was dissected off, the sciatic nerve being left attached to the muscle. This muscle was placed in a watch-crystal containing a solution of aconitine, 1-5 of a milligramme to the cubic centimetre; the nerve being suspended out of the fluid. In another glass the sciatic nerve of a second leg prepared as the first, was immersed in a solution of the same strength, while the muscle was kept from contact with the fluid; the preparations were covered with a dampened bell-glass; after a time, the nerve in the first preparation had completely lost its excitability; whilst in the second preparation the muscles responded promptly to the excitation of the nerve. Aconite then destroys the motor power of a nerve by acting through its peripheric terminations.

*Third experiment:* Before poisoning the animal, the circulation of one of the posterior members was cut off. All the motor nerves which received the poisoned blood, lost their physiological power; whilst the nerves of the leg, in which the circulation was arrested, continued perfectly excitable. It was observed that sensibility remained as long as muscular excitation through the motor nerves was possible.

These experiments made after the method of Claude Bernard, seem to establish the fact that in small doses the physiological effects of aconitine are analogous to those of curarine. Aconitine like curare exerts its first effects on the motor nerves.

Finally we made another experiment which, at first, puzzled us: We had injected into a frog one milligramme of aconitine, that is, a dose twenty times as large as those used in the former experiments; we were greatly astonished to observe that the animal retained, for a very long time, the excitability of the motor nerves, and that convulsive movements were continually made; but afterward, in opening the thorax, we recognized that the ventricle of the heart was completely stilled, while the auricles alone were feebly contracting. We then conceived the idea, that the posion, when thus administered in a strong dose, was competent to arrest primitively the heart's action; a result which would have the effect of preventing the absorption of the poison [through the circulation]. Experiment fully coroborated this hypothesis. A frog was disposed under the microscope for the examination of the interdigital membrane: one milligramme of aconitine was thrown under the skin; at the expiration of one and a half minutes, the circulation in the arteries was considerably abated; in three minutes it was completely arrested. Upon opening the thorax, the ventricle was motionless. The nerves of the brachial plexus were found excitable, but a little less so than the lumbar nerves, which retained almost entirely their normal excitability. The action of the heart being arrested, the poisoning could only take place by imbibition. With mammals, the toxic phenomena of aconitine are much more rapid, and much less easy of analysis. We have injected into a rabbit one milligramme of aconitine, and then kept up artificial respiration; at the end of half an hour the sciatic nerve no longer determined contractions in the muscles of the leg, which nevertheless preserved their contractility.

